# Organophosphate Esters and Polybrominated Diphenyl Ethers in Vehicle Dust: Concentrations, Sources, and Health Risk Assessment

**DOI:** 10.3390/toxics12110806

**Published:** 2024-11-07

**Authors:** Junji Wang, Jianzai Lin, Xi Zhang, Qinghong Zeng, Zhu Zhu, Siyuan Zhao, Deyan Cao, Meilin Zhu

**Affiliations:** 1 School of Public Health, Ningxia Medical University, Yinchuan 750004, China; wjjeee2022@163.com (J.W.); omfyer@163.com (J.L.); 18847020141@163.com (Q.Z.); m15023462013@163.com (Z.Z.); deverezhao98@163.com (S.Z.); jolly2025@163.com (D.C.); 2 Key Laboratory of Environmental Factors and Chronic Disease Control, Ningxia Medical University, Yinchuan 750004, China; 3 School of Basic Medical Sciences, Ningxia Medical University, Yinchuan 750004, China; 18784042561@163.com

**Keywords:** vehicle dust, organophosphate esters and polybrominated diphenyl ethers, PCA, PMF, health risk assessment, Monte Carlo simulation

## Abstract

Background: The primary flame retardants in vehicles, organophosphates (OPEs) and polybrominated diphenyl ethers (PBDEs), volatilize and accumulate in the enclosed vehicle environment, posing potential health risks. Amidst the rising number of vehicles, the scrutiny of persistent organic pollutants like OPEs and PBDEs in vehicles is increasing. This study investigates occupational and nonoccupational population exposure to specific OPEs (TnBP, TBOEP, TEHP, TCEP, TCiPP, TDCiPP, TPhP, EHDPP) and PBDEs (BDE-28, BDE-47, BDE-99, BDE-100, BDE-153, BDE-154, BDE-183, BDE-209) in vehicle dust. Methods: Data on OPEs and PBDEs in vehicle dust were sourced from PubMed and Web of Science. We applied PCA and PMF to identify pollutant sources and assessed health risks using the hazard index (HI) and carcinogenic risk (CR) methods. Monte Carlo simulations were conducted for uncertainty analysis, evaluating variable contributions to the results. Results: The predominant OPE in dust samples was TDCiPP (mean value: 4.34 × 10^4^ ng g^−1^), and the main PBDE was BDE-209 (mean value: 1.52 × 10^4^ ng g^−1^). Potential sources of OPEs in vehicle dust include polyvinyl chloride (PVC) upholstery, polyurethane foam (PUF) seats, electronics, carpet wear, hydraulic oil, and plastic wear in the brake system. PBDE sources likely include automotive parts, PVC upholstery, seats, carpets, and electronics. The 90th percentile HI and CR values for occupational and nonoccupational populations exposed to OPEs and PBDEs indicate that the noncarcinogenic and carcinogenic risks are relatively low. A sensitivity analysis showed that the pollutant concentration, time in the vehicle, exposure frequency, and duration significantly influence health risks. Conclusions: The health risks to both occupational and nonoccupational populations from exposure to OPEs and PBDEs in vehicle dust are relatively low.

## 1. Introduction

PBDEs are a type of brominated flame retardant that are considered persistent organic pollutants. They are often utilized in a variety of plastic products, including textiles, electronics, automobiles, and building materials, due to their exceptional flame-retardant qualities and minimal impact on material properties. However, the environmental risks associated with PBDEs have led to the classification of penta-BDE and octa-BDE as persistent organic pollutants by the Stockholm Convention in 2009, with deca-BDE also being added to the list of priority controlled pollutants in 2017 [1,2,3]. Organophosphorus flame retardants are a type of organic compound that has been increasingly used as an alternative to brominated flame retardants due to their environmental restrictions and phase-out [4]. OPEs are utilized as plasticizers and defoamers in a wide range of household and industrial products, including upholstery, floor polishes, paints, electronic equipment, and personal care items. Because of their properties, OPEs can easily enter and contaminate the environment through volatilization, dissolution, and physical abrasion during production and use. Research has indicated that PBDEs and OPEs can be found in microenvironmental dust worldwide, including in countries such as China, Japan, Germany, Egypt, New Zealand, and the United Kingdom [5,6,7,8,9,10], primarily in indoor and automotive microenvironmental dust [11,12]. Similarly, the use of OPEs is restricted by the European Union, with ten congeners having usage restrictions under the REACH regulation or being listed as restricted substances in Annex XVII of the REACH regulation. Individual states in the United States have also introduced bans or restrictions on the use of OPEs in consumer products [13,14].

Organophosphorus and brominated flame retardants are incorporated into products based on their physicochemical properties rather than being chemically bound to materials. This mode of incorporation allows them to be released from products into the environment and adhere to dust, particles, and material surfaces due to their low saturated vapor pressure. Exposure to these flame retardants can occur through oral ingestion, skin contact, and inhalation [15], with dust ingestion being the primary pathway [5]. According to toxicological studies, certain substances, such as polybrominated diphenyl ethers and organophosphates, have been classified as environmental endocrine disruptors with harmful effects on health, including carcinogenic, genotoxic, and reproductive toxicity effects [16]. When consumed at high doses, BDE-209 can lead to decreased serum thyroid hormone levels, thyroid function status alterations, and organ structure damage, which can result in reproductive and developmental toxicity [17]. Exposure to BDE-47 can have negative effects on adult male mice, including reduced sperm motility and viability and impaired learning abilities and memory retention [18]. Furthermore, BDE-99 is known for its high toxicity as a flame retardant, which primarily affects the nervous system by causing memory loss and decreased thyroid hormone levels in the body [19]. Research has indicated that chemicals such as TCEP, TCiPP, TDCiPP, and TBOEP may have harmful effects on both human health and embryonic growth [20]. Exposure to TDCiPP has been linked to adverse effects on gallbladder development and a decrease in plasma thyroxine levels [21]. Moreover, acute exposure to TPhP has been found to impair neurodevelopment in zebrafish larvae and decrease acetylcholinesterase activity [22]. Finally, TnBP has been shown to cause oxidative stress, DNA damage, and apoptosis [23]. It is important to be aware of the potential risks associated with these chemicals and take appropriate precautions to minimize exposure.

Over the years, researchers have conducted extensive studies on flame retardants found in indoor dust, predominantly originating from offices, homes, schools, and cars [24,25,26,27]. Studies have revealed that the concentration of flame retardants in cars is ten times greater than that in other indoor environments, such as homes and offices [7]. This is mainly due to the fact that pollutants are more likely to accumulate when there is limited space in the car and poor air mobility. And the car is susceptible to exposure in summer, and the temperature in the car can reach more than 65 °C [28]. Elevated temperatures contribute to the release of flame retardants from car interiors, dashboards, and seats into gas or particle phases, leading to their presence in car dust [29,30]. Additionally, flame retardants from other indoor environments such as homes or offices may be carried into the vehicle. Unfortunately, there are few studies on the noncarcinogenic and carcinogenic risks for occupational populations exposed to OPEs and PBDEs in automobile dust. It is crucial to conduct further studies to identify the noncarcinogenic and carcinogenic risks faced by individuals in professional occupations who spend approximately two-thirds of their time inside a vehicle [7].

Against this backdrop, this paper reviews the available literature and aims (1) to investigate the levels of and correlation between OPE and PBDE pollution in automobile dust; (2) to identify the sources of OPE and PBDE pollution in automobile dust using PCA and PMF models; (3) to assess the health risks associated with occupational and nonoccupational exposure to automobile dust using the hazard index (HI) and carcinogenic risk (CR) methods; and (4) to conduct a Monte Carlo simulation for uncertainty analysis, evaluating the contribution of each variable to noncarcinogenic and carcinogenic outcomes.

## 2. Methods

### 2.1. Data Processing

To ensure the accuracy and authority of our data sources, this study retrieved articles from internationally recognized databases (PubMed and Web of Science), using a census method for data collection. Our data retrieval period spanned from 1 January 2010 to 1 November 2023, during which we searched for the literature on the topic of “automobile dust” and obtained 1337 results. We then conducted an advanced search using keywords such as “automobile dust”, “flame retardants”, “polybrominated diphenyl ethers”, and “organophosphorus flame retardants”. Ultimately, we obtained 27 studies on pollutant detection with clear sample types and experimental results; the data sources are shown in Table S1. To facilitate risk assessment, we excluded compounds with limited data and identified eight organophosphorus flame retardants (TnBP, TBOEP, TEHP, TCEP TCiPP, TDCiPP, TPhP, and EHDPP) and eight polybrominated diphenyl ethers (BDE-28, BDE-47, BDE-99, BDE-100, BDE-153, BDE-154, BDE-183, and BDE209) for further data collection and processing.

We attempted to use the results of specific individual samples in these studies. If the source did not disclose the individual test results for each sample and only provided average test sample results, we considered the average as a representative result of a single sample. For sample values below the detection limit, they were considered to be half of the detection limit according to the reference literature [31]. We used Microsoft Excel (2021, 64-bit) to calculate the mean value of the collected sample results. When the range of sample results was published in papers, we adopted both the minimum and maximum values as the corresponding ranges. Minimum detection limits (MDLs) were determined to calculate the uncertainty in the PMF source analysis.

### 2.2. Statistical Methods

This study employed statistical methods to investigate the correlation between and sources of organophosphate esters and polybrominated diphenyl ethers in vehicle interiors. First, Spearman’s correlation analysis was utilized to examine the relationship between organophosphate esters and polybrominated diphenyl ethers. Correlation analysis quantifies the degree of association between variables, with a strong correlation indicating similar properties or origins. Next, two commonly used multivariate techniques—principal component analysis (PCA) and positive matrix factorization (PMF)—were applied to explore their sources. The primary reason for employing both PCA and PMF models was to achieve a more comprehensive analysis of the data. PCA can rapidly identify the main components within the data, while PMF can further quantify the contribution of each pollution source. The combined use of these two methods allows for a more thorough analysis of pollution sources. PCA is a method that recombines initially correlated variables into a new set of linearly independent variables through orthogonal transformation, followed by dimensionality reduction, to compare the contribution rates of each variable [32]. The PMF method is widely employed for quantifying the composition and profiles of various environmental matrices, including water, the atmosphere, dust, and sediment.

In Equation (1), *X* denotes *n* measured samples and m chemicals, and *F* is the matrix of *p* chemical profiles. The *G* matrix is the contribution of each factor to any given sample, and *E* is the residual matrix:

(1) X=GF+E

To reduce the rotational degrees of freedom, the PMF uses the least squares method by iteratively fitting the data until the fitting parameter *Q* is minimized.

(2) $$Q=\sum_{i=1}^{m}\sum_{j=1}^{n}{\left(\frac{e_{i\dot{j}}}{\delta_{ij}}\right)}^{2}$$

where *Q* is the sum of the squares of the differences (*e_ij_*) between the observations (*X*) and the model (*GF*), weighted by the measurement uncertainty (*σ_ij_*), and the uncertainty for a single sample is calculated using the error fraction and the method detection limit (*MDL*). If the concentration is less than or equal to the *MDL*, the uncertainty (*σ_ij_*) is calculated using the fixed fraction of the *MDL* (Equation (3)) [33].

(3) $$\delta_{ij}=\frac{5}{6}\times MDL$$

When the concentration is higher than the *MDL*, a fraction based on the user-supplied concentration and the *MDL* is calculated (Equation (4)).

(4) $$\delta_{ij}=\sqrt{{\left(Error\;fraction\times C\right)}^{2}+{\left(0.5\times MDL\right)}^{2}}$$

### 2.3. Risk Assessment

#### 2.3.1. Exposure Assessment

The objective of conducting an exposure assessment is to determine the extent of exposure that the target population has to a particular substance. The target population in this study consists of adults in both occupational and nonoccupational groups. The occupational group refers to drivers who are exposed to the interior of vehicles for extended periods due to their work. The nonoccupational group refers to the general public, such as those who are exposed to the interior of vehicles during their daily commutes. The focus of this study was to evaluate the exposure of individuals to OPEs and PBDEs found in dust through three primary routes: hand-to-mouth ingestion, inhalation, and skin contact. To achieve this goal, an assessment model recommended by the US EPA was utilized [34]. Additionally, this study assumed a 100% absorption rate where substances entering human bodies are completely absorbed [5,35,36,37].

The average daily dose (*ADD*) (mg/kg/day) of contaminants in the dust absorbed through the three routes of ingestion, dermal contact, and inhalation was estimated using Equations (5)–(7) [38]. Where *C* (mg kg^−1^) is the concentration of OPEs and PBDEs in dust, *R_ing_* (g day^−1^) is the ingestion rate, *R_inh_* (m^3^ day^−1^) is the inhalation rate, *ET* is the time spent in the vehicle in a day, and *EF* is the frequency of exposure. Here, *ED* is the duration of exposure, *PEF* (1.39 × 10^9^ m^3^ kg^−1^) is the particulate emission factor, *SA* (cm^2^) is the skin exposure area, *DA* (mg cm^−2^) is the skin absorption factor, *AF* is the skin attachment factor, *BW* (kg) is body weight, and *AT* (days) is the averaging time. Table S2 lists the specific values of the above parameters.

(5) $${ADD}_{ingestion}=C\times \frac{R_{ing}\times ET\times EF\times ED}{BW\times AT}\times CF$$

(6) $${ADD}_{inhalation}=C\times \frac{R_{inh}\times ET\times EF\times ED}{PEF\times BW\times AT}\times CF$$

(7) $${ADD}_{dermal}=C\times \frac{SA\times DA\times AF\times ET\times EF\times ED}{BW\times AT}\times CF$$

#### 2.3.2. Noncarcinogenic Risk Assessment

The noncarcinogenic risk that a chemical substance may cause is expressed in terms of a hazard quotient (*HQ*) and a hazard index (*HI*), which is equal to the ratio of daily exposure to the chronic reference dose (RfD) for non-carcinogenicity. When the *HQ* value is less than or equal to 1, exposure is not likely to be associated with adverse health effects; however, when the *HQ* value is greater than 1, the potential for adverse effects increases. Given the wide variety of OPE and PBDE congeners with different levels of toxicity, the United States Environmental Protection Agency (US EPA) has proposed reference doses for six organophosphate isomers (TNBP, TBOEP, TCEP, TCIPP, TDCIPP, and TPHP) and four PBDE congeners (BDE-47, BDE-99, BDE-153, and BDE 209) as RfD values; the RfD values are listed in the Supplementary Materials. The formula for calculating the noncarcinogenic risk is as follows:

(8) $$HQ=\frac{ADD}{RfD}$$

(9) $$HI=\sum_{i=1}^{n}{HQ}_{i}$$

#### 2.3.3. Carcinogenic Risk Assessment

The carcinogen risk (*CR*) is the likelihood that an individual will develop any type of cancer as a result of lifetime exposure to a carcinogenic chemical. The health risk characterized by carcinogen risk is based on a slope factor (*SF*). Slope factors are used in carcinogen risk assessments to estimate an individual’s lifetime probability of developing cancer as a result of exposure to a specific carcinogen. Risks at *CR* values above 1 × 10^−4^ are generally considered relatively high, risks at *CR* values below 1 × 10^−6^ are considered negligible, and risks at *CR* values between 1 × 10^−4^ and 1 × 10^−6^ are relatively low. The formula for calculating the carcinogenic risk is as follows:

(10) CR=ADD×SF

#### 2.3.4. Probabilistic Assessment and Sensitivity Analysis

In the uncertainty analysis of the health risk assessment, there are two primary components: determining the probability outcomes and evaluating the contribution of each variable to the results. First, we employed the Monte Carlo simulation method to simulate exposure factors (such as chemical concentration, daily intake, exposure frequency, and body weight) by utilizing the Anderson-Darling test and chi-square test to ascertain the most suitable probability distribution type. Stable exposure distribution results were obtained through 10,000 iterations, and different magnitude values (e.g., 10th percentile, 50th percentile, and 90th percentile) of the exposure distribution results were utilized. A sensitivity analysis was primarily employed to assess the contribution of each exposure factor to the results. Initially, rank correlation coefficients between exposure factors and health risks were determined using probability estimation methods. Subsequently, contributions from each variable were calculated by squaring their variances. Finally, to generate a sequence of contributing variables uniformly expressed as percentages, positive values indicated a positive correlation between exposure coefficients and health risks, while negative correlations implied an inverse relationship.

### 2.4. Data Analysis

The mean of the collected sample results was calculated using Microsoft Excel (64-bit, 2021 version). Data correlation and PCA were conducted using SPSS (version 20.0). EPA PMF 5.0 software was utilized for the PMF analysis [39]. Crystal Ball software (version 11.1.3.0.000, 64-bit) was employed for the probability assessment and sensitivity analysis. Origin 2021 version was used for data visualization.

## 3. Results and Discussion

### 3.1. OPEs and PBDEs in Vehicle Dust

Table 1 displays the resulting figures after the data were processed. The analysis revealed that the dust in the vehicle contained 25.8–2.15 × 10^6^ ng g^−1^ and 0.17–3.03 × 10^5^ ng g^−1^ OPEs (∑_8_OPEs) and PBDEs (∑_8_PBDEs), respectively, with average concentrations of 9.45 × 10^3^ ng g^−1^ and 1.31 × 10^3^ ng g^−1^. The mean concentration of TDCiPP in the organophosphate ester group was 4.34 × 10^4^ ng g^−1^, while the mean concentration of BDE-209 in the polybrominated diphenyl ether group was 1.52 × 10^4^ ng g^−1^. The two congeners with the lowest contents were TnBP (mean value: 1.91 × 10^2^ ng g^−1^) and BDE-28 (mean value: 11.4 ng g^−1^).

The concentrations of OPEs and PBDEs in automobile dust from various countries are shown in Tables S3 and S4. Among these, South Africa has the highest concentration of OPEs (1.44 × 10^5^ ng g^−1^) in vehicle dust, while Nigeria has the lowest (278 ng g^−1^); the United States has the highest concentration of PBDEs (4.06 × 10^4^ ng g^−1^) in vehicle dust, with Thailand having the lowest (50.8 ng g^−1^). These variations may be attributed to the differing usage patterns of OPEs across different countries [40,41]. The primary contributors to OPEs in automobile dust in this study were TDCiPP, TCiPP, and TBOEP, which aligns with findings from other studies in Greece and Japan, where TDCiPP and TCiPP were the dominant compounds [6,42] and TBOEP and TPHP also made significant contributions [43]. In line with the findings of previous studies, BDE-209 once again had the highest range and mean value of all the studied congeners, as has been observed in previous research [32,44]. This may be attributed to the earlier inclusion of penta-BDE and octa-BDE in the ban list than deca-BDE. Additionally, compared with other PBDE congeners, BDE-209 has a greater affinity for solid particle surfaces and lower volatility, which contributes to its longer residence time in automotive dust. Variations in flame retardant content within the dust can be linked to fire safety regulations across different countries, such as the EU and the US, which banned or limited the production and use of deca-BDE in 2008 and 2013, respectively. However, many nations, including China, have yet to propose equivalent restrictions [45]. Furthermore, the content of flame retardants in dust can also be influenced by the age, usage, and wear and tear of the vehicle, as well as by environmental factors such as high temperatures and limited ventilation.

### 3.2. Spearman’s Correlation Between OPEs and PBDEs in Vehicle Dust

**Figure 1:**
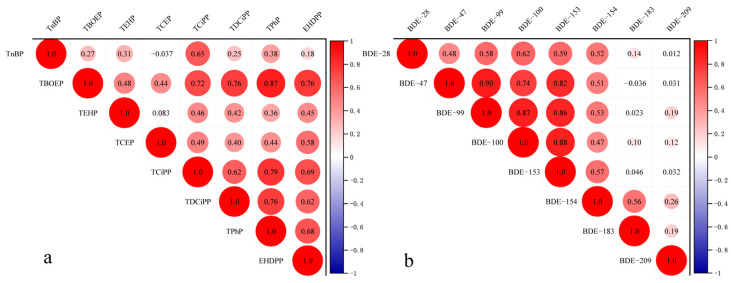
Heatmap of the correlation between OPEs and PBDEs. (**a**) OPEs (**b**) PBDEs.

Correlation analysis conveniently facilitates the identification of associations between target substances. Prior to conducting the analysis, we assessed the normality of the data and found a weak adherence to a normal distribution. Consequently, Spearman’s correlation coefficient was employed for the bivariate correlation analysis of OPEs and PBDEs in vehicle dust, providing direct insights into pollution source similarities for each OPE and PBDE. The results of this analysis are presented in Figure 1.

Figure 1 shows that there was a highly significant positive correlation between TBOEP and TCiPP (*r* = 0.72, *p* ≤ 0.001), TDCiPP (*r* = 0.76, *p* ≤ 0.001), TPhP (*r* = 0.87, *p* ≤ 0.001), and EHDPP (*r* = 0.76, *p* ≤ 0.001). There was also a highly significant positive correlation between TPhP and TCiPP (*r* = 0.79, *p* ≤ 0.001) and between TPhP and TDCiPP (*r* = 0.76, *p* ≤ 0.001). Additionally, there was a significant positive correlation between TnBP and TCiPP (*r* = 0.65, *p* ≤ 0.001). BDE-47 showed an extremely significant positive correlation with BDE-99 (*r* = 0.90, *p* ≤ 0.001), BDE-100 (*r* = 0.74, *p* ≤ 0.001), and BDE-153 (*r* = 0.82, *p* ≤ 0.001). Similarly, there was an extremely significant positive correlation between BDE-99 and both BDE-100 (*r* = 0.87, *p* ≤ 0.001) and BDE-153 (*r* = 0.86, *p* ≤ 0.001). Finally, there was an extremely significant positive correlation between the BDE-100 concentration and the BDE-153 concentration (*r* = 0.88, *p* ≤ 0.001).

Highly correlated congeners likely share a common source of pollution, while congeners with weak and difficult-to-classify correlations may have similar sources of contamination. TDCiPP and TCiPP are highly likely to originate from the same source, as they are commonly regarded as substitutes for penta-BDE mixtures [6]. TBOEP and TPhP are frequently utilized as plasticizers in automotive dashboards and electronic instruments. BDE-47, BDE-99, BDE-100, BDE-153, and BDE-154 constitute major components of penta-BDE and presumably share the same origin. The limited correlation between BDE-209 and other congeners could be attributed to its decomposition under high temperatures or light exposure [32]. Although certain congeners exhibit significant correlations with each other, further categorization of their sources is necessary.

### 3.3. Principal Component Analysis (PCA) of OPEs and PBDEs in Vehicle Dust

**Figure 2:**
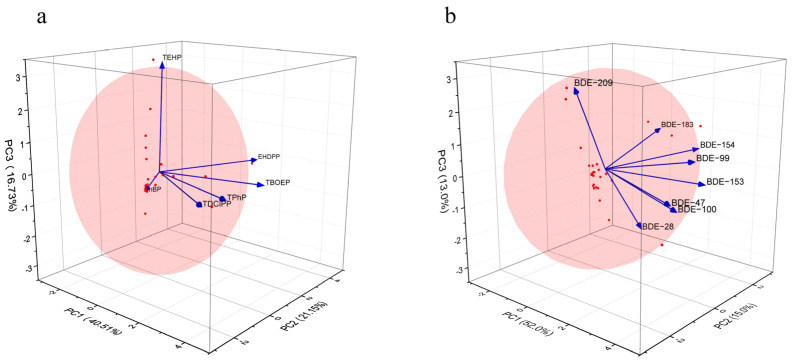
Principal component analysis of OPEs and PBDEs in vehicle dust. (**a**) OPEs (**b**) PBDEs. Red spots mean sample point.

PCA was conducted to investigate the sources of OPEs and PBDEs in car dust. Due to the requirement that the result of the Kaiser–Meyer–Olkin test should be greater than 0.6 for factor analysis, two congeners with low common factor variances (TCiPP and TCEP) were excluded from the data analysis, while Bartlett’s sphericity test was passed. In the analysis of OPEs, factor 4 in the rotated factor loading had an eigenvalue of 0.97, which is slightly less than one. However, grouping OPEs with significant source differences into one factor would occur if only three factors were determined. Therefore, it was necessary to determine four factors that collectively account for 95.06% of the variance. Including factor 4 significantly improves the proportion of total variance explained, and the scree plot shows a clear inflection point at factor 4. In the analysis of PBDEs, the Kaiser–Meyer–Olkin test result was greater than 0.6, and Bartlett’s test of sphericity was significant. For PBDEs, three factors were identified, accounting for a total of 80.07%. Tables S5 and S6 present the explained values of each factor variance. Figure 2 shows the three-dimensional load diagram for each factor of the OPEs and PBDEs. The scree plots for OPEs and PBDEs are provided in the Supplementary Material.

OPE factor 1 explained 40.51% of the total variance and was strongly positively correlated with TPhP, TDCiPP, and TBOEP. TPhP, TDCiPP, and TBOEP are the main flame retardants used in polyurethane foam (PUF), particularly in automotive seat cushions and other decorative materials [46,47]. Additionally, TPhP is an alternative to penta-BDE [48,49]. Studies have shown that residential areas with foam seats and carpets have higher average concentrations of TCiPP and TDCiPP [40,50]. Therefore, the potential source of factor 1 could be car seats. OPE factor 2 explained 21.15% of the total variance, was strongly positively correlated with EHDPP, and was moderately positively correlated with TBOEP. EHDPP is commonly added to polyvinyl chloride (PVC), rubber, and polyurethane materials [51]. Thus, factor 2 represents the release of the PVC and PUF used in car interiors. OPE factor 3 explained 16.73% of the total variance and was strongly positively correlated with TnBP. TnBP is widely used in hydraulic oil, defoamers, lubricants, etc. [52], suggesting that factor 3 may originate from the release of hydraulic oil from braking systems inside cars. OPE factor 4 explained 16.67% of the total variance and was strongly positively correlated with TEHP, which serves as a plasticizer for PVC automotive floor mats [46]. Thus, factor 4 might be from worn car carpets. Table S2 shows the rotational component matrix of OPEs.

Factor 1 of the PBDEs explained 49.58% of the total variance and was strongly positively correlated with BDE-47, BDE-99, BDE-100, and BDE-153 and moderately positively correlated with BDE-154. These compounds are components of penta-BDE, which are commonly used in polyurethane foam, sponge materials, and interior decoration [48]. Studies have found higher concentrations of PBDEs in rooms with heavily worn carpets [53]. Therefore, it is likely that factor 1 is related to car seats and carpets. Factor 2 of the PBDEs explained 16.85% of the total variance, was strongly positively correlated with BDE-154 and BDE-183, and was weakly correlated with BDE-153 and BDE-99. The compounds BDE-183, BDE-154, and BDE-153 are components of octa-BDE formulations widely used in the production of acrylonitrile–butadiene–styrene (ABS) polymers for the automotive industry, electrical instrument industry, and mechanical industry [54]. Therefore, the potential source for factor 2 could be automotive parts. Factor 3 of the PBDEs explained 13.67% of the total variance, was strongly positively correlated with BDE-209, and was weakly correlated with BDE-99. BDE-209 is a major component of deca-BDE formulations, which are commonly used in plastics and electronic devices [55]. Therefore, the potential sources for factor 3 could be car electronics or PVC interiors. Table S3 shows the rotational component matrix of PBDEs.

### 3.4. Positive Matrix Factorization (PMF) of OPEs and PBDEs in Vehicle Dust

**Figure 3:**
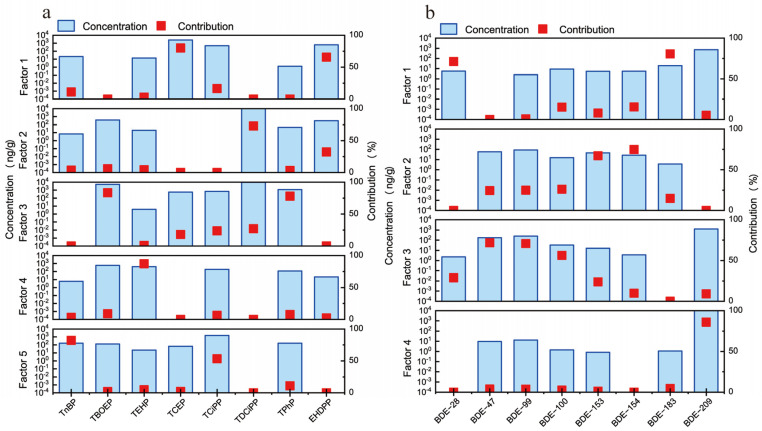
Source profile and source contribution of OPEs and PBDEs from PMF analysis. (**a**) OPEs (**b**) PBDEs.

The contributions of each factor are shown in Figure 3. We identified five major factors that contribute to OPEs. Factor 1 had high levels of TCEP and EHDPP, accounting for 79.85% and 65.52% of the contributions, respectively. TCEP is commonly used in polyurethane foam, textiles, and plastics [56], while EHDPP is often added to PVC materials [51]. Based on this, we labeled factor 1 as the contribution of automotive interior PVC and PUF. Factor 2 had a greater contribution to TDCiPP, which is typically used in PUF for decorative materials such as car seat foams [56]. Therefore, factor 2 represents the release of PUF in car seats. Factor 3 included TBOPE and TPhP, with contribution rates of 82.29% and 78.01%, respectively. TBOPE is commonly used as a structural material for car dashboards or other plastic devices [57]. TPhPs are used in electronic devices, PVCs, adhesives, casting resins, styrene-based resins, and engineering thermoplastics [58]. Hence, the source of factor 3 emissions is electronic equipment. Factor 4 had a greater contribution to the TEHP at 87.04%. It is used in cellulose, PVC, rubber, paints, and coatings, as well as in polyurethane foam [46]. TEHP is commonly used as a plasticizer for car floor mats made of PVC, thus contributing to carpet wear inside the vehicle. Factor 5 had high TnBP and TCiPP loads of 83.10% and 53.28%, respectively. TnBP is widely used in hydraulic oils, defoamers, metal complexing agents, coatings, and plastics [52]. TCiPP is extensively used in resins, plastics, and cellulose [58]. Factor 5 was identified as hydraulic oil and plastic wear from the car’s braking system.

**Figure 4:**
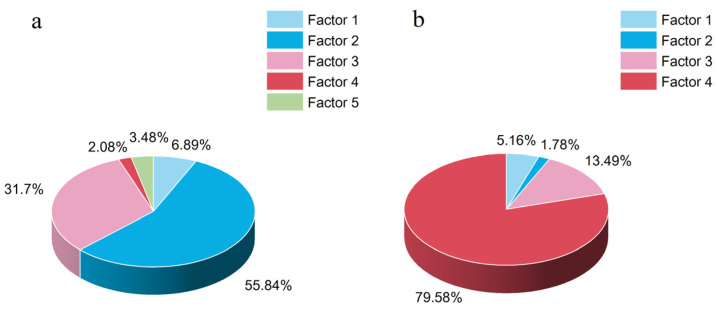
Source contributions from pollution sources calculated through PMF analysis. (**a**) OPEs and (**b**) PBDEs.

We identified four main factors for PBDEs. Factor 1 was primarily composed of BDE-28 and BDE-183, with BDE-183 being mainly used in the plastic components of automobiles, textiles, and electronic devices [59]. The industrial manufacturing and use of these materials may result in the emission of BDE-183. Therefore, the source of factor 1 is car parts containing octa-BDE formulations. Factor 2 was dominated by BDE-154 and BDE-153, with lower contributions from BDE-47, BDE-99, and BDE-100. Factor 3 was mainly composed of BDE-47, BDE-99, and BDE-100, with lower contributions from BDE-28 and BDE-153. Factors 2 and 3 were loaded with Tris to form hexa-BDE as the main component of penta-BDE [60]. It has been reported that PUR foam in vehicles and buildings accounts for 90% to 95% of the total usage of penta-BDE, with a small amount used in PVC materials, etc. [61]. Penta-BDE is mainly applied to textiles and PUR foam products, with a focus on BDE-47 and BDE-99. Therefore, the source of factor 2 is PVC interiors, and factor 3 is categorized as automotive seats and carpets. Factor 4 was primarily composed of BDE-209, which is a crucial ingredient in commercial deca-BDE formulations. It accounts for 92% to 97% of the total composition. Deca-BDE is widely used in various industries, including electronics, rubber, textiles, and plastics. It is particularly suitable for synthetic materials such as high-impact polystyrene (HIPS), polyethylene (PE), polypropylene (PP), ABS resin, and rubber fibers [59]. This factor indicates the contribution of electronic devices used in vehicles. The distribution of source contributions is shown in Figure 4.

### 3.5. Exposure Assessment and Health Risk Evaluation

#### 3.5.1. Exposure Assessment

The exposure levels of OPEs and PBDEs in both occupational and nonoccupational populations were calculated through three primary routes: inhalation, ingestion, and dermal contact. Our findings, which can be found in Table 2, indicate that the average daily exposure of the occupational population was greater than that of the nonoccupational population. The mean daily exposure to ∑OPEs via ingestion (occupational populations: 1.49 × 10^−5^ mg kg^−1^ day^−1^; nonoccupational populations: 2.25 × 10^−6^ mg kg^−1^ day^−1^) and dermal absorption (occupational populations: 2.47 × 10^−6^ mg kg^−1^ day^−1^; nonoccupational populations: 3.72 × 10^−7^ mg kg^−1^ day^−1^) was 6~7 orders of magnitude greater than inhalation exposure (occupational populations: 4.40 × 10^−12^ mg kg^−1^ day^−1^; nonoccupational populations: 6.62 × 10^−13^ mg kg^−1^ day^−1^). Similarly, the average daily exposure to ∑PBDEs through ingestion (occupational populations: 3.12 × 10^−6^ mg kg^−1^ day^−1^; nonoccupational populations: 4.69 × 10^−7^ mg kg^−1^ day^−1^) and dermal absorption (occupational populations: 2.88 × 10^−7^ mg kg^−1^ day^−1^; nonoccupational populations: 4.33 × 10^−8^ mg kg^−1^ day^−1^) was 6~7 orders of magnitude greater than that resulting from inhalation exposure (occupational populations: 9.17 × 10^−13^ mg kg^−1^ day^−1^; nonoccupational populations: 1.38 × 10^−13^ mg kg^−1^ day^−1^). For both groups, the exposure levels of OPEs and PBDEs were much lower than the oral RfD, which is consistent with other research findings [62,63,64]. The ingestion of vehicle dust was found to be the primary source of exposure for both occupational and nonoccupational populations, accounting for 84% of exposure to OPEs, and 16% was accounted for by dermal contact. Similarly, the ingestion of vehicle dust was the main contributor to exposure to PBDEs, accounting for 92% of the total exposure, with dermal contact contributing to 8% of the total exposure. The inhalation of vehicle dust had an insignificant effect on both groups, which is consistent with previous research findings [50,65]. Additionally, the daily average exposure of occupational individuals is greater than that of nonoccupational individuals, with higher levels of organophosphate exposure compared to polybrominated diphenyl ether. This is due to the longer duration of exposure to vehicles for occupational individuals and the gradual replacement of brominated flame retardants with organophosphorus flame retardants in recent years [66].

#### 3.5.2. Noncarcinogenic Exposure

Table S7 provides a summary of the HQ and HI values for the three exposure pathways. Notably, TDCiPP had significantly higher HQ values than the other similar substances studied, with values ranging from one to two orders of magnitude greater. The order of contribution for OPEs was TDCiPP (72%) > TBOEP (16%) > TCEP (6%) > TCiPP (3%) > TPhP (2%) > TnBP (~0%), while for PBDEs, it was BDE-99 (48%) > BDE-47 (30%) > BDE-209 (17%) > BDE-153 (6%). TDCiPP and BDE-99 have the highest HI values, which is consistent with the risk analysis of dust inside cars [44,67]. The results indicate that for both occupational and nonoccupational populations, the HI values for exposure to OPEs and PBDEs in vehicle dust through three pathways are all less than one, suggesting that exposure is not likely to be associated with adverse health effects. The primary mode of exposure was ingestion, with inhalation exposure posing a negligible risk compared to ingestion and dermal contact.

#### 3.5.3. Carcinogenic Exposure

Table S7 includes the CR associated with the three exposure pathways. As the US EPA only provided updated slope cancer factors (SFs) for five congeners (TnBP, TEHP, TCEP, TDCiPP, and BDE-209), we assessed the cancer risk for only these five congeners. Our findings indicate that the CR values for all three exposure routes were less than 1 × 10^−6^, revealing that the carcinogenic risk from exposure was almost negligible. The reference doses and carcinogenic slope values for OPEs and PBDEs were derived from short-term animal experiments [68]. However, our assessment focused on the long-term exposure of humans to toxic substances and was limited by the lack of SF values for other congeners. The actual carcinogenic risk may be greater than what we currently evaluate. The carcinogenic risks associated with ingestion and dermal contact exposure are much greater than those associated with inhalation exposure, indicating a greater risk through these two pathways of exposure.

#### 3.5.4. Probability Assessment

**Figure 5:**
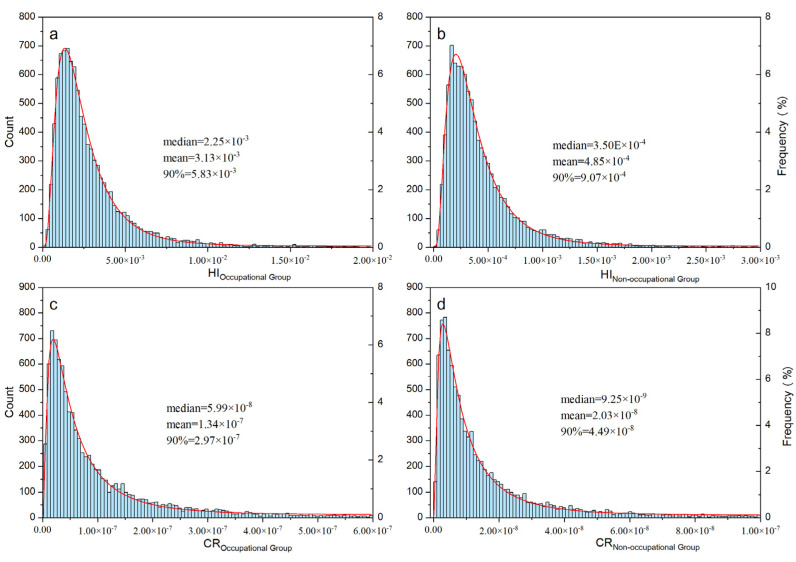
Probabilistic health risk assessment for OPEs and PBDEs in vehicle dust: noncarcinogenic risks and carcinogenic risks. (**a**) HI of occupational group. (**b**) HI of non-occupational group. (**c**) CR of occupational group. (**d**) CR of non-occupational group.

A Monte Carlo simulation was used to evaluate the risk of OPE and PBDE exposure through ingestion, absorption, and dermal contact in both occupational and nonoccupational populations. Figure 5 displays the results, indicating that the occupational group is at a greater risk of noncarcinogenic and carcinogenic exposure than the nonoccupational population. The 90th percentile HIs for both groups are less than one, indicating that exposure is not likely to be associated with adverse health effects. Furthermore, the 90th percentile CRs for both groups are less than 1 × 10^−6^, also suggesting that exposure is not likely to be associated with carcinogenic risks. Therefore, both occupational and nonoccupational populations face no significant risk of noncarcinogenic or carcinogenic exposure to OPEs and PBDEs. Table S8 lists the information for each distribution and the different percentiles.

#### 3.5.5. Sensitivity Analysis

**Figure 6:**
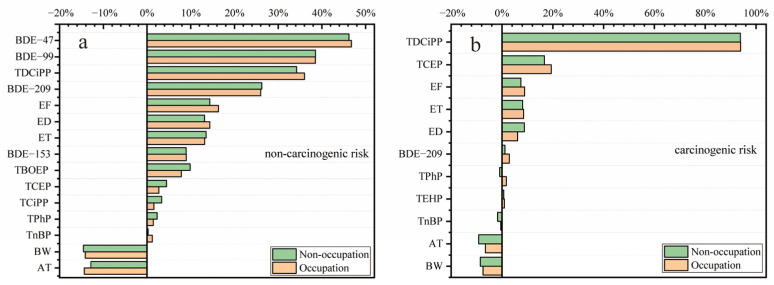
Sensitivity analysis of the carcinogenic risk of OPE and PBDE exposure from OPEs and PBDEs in vehicle dust. (**a**) Non-carcinogenic risk (**b**) Carcinogenic risk.

A sensitivity analysis was used to measure the uncertainty in the parameters used for the probabilistic risk assessment. The results are shown in Figure 6. The main factors that increase the risk of noncarcinogenic pollution are the concentrations of BDE-47 (46.18–46.74%), BDE-99 (38.51–38.56%), TDCiPP (34.21–36.03%), and BDE-209 (26.00–26.23%). The primary factor that increased the carcinogenic risk was the concentration of TDCiPP (93.83–93.92%). The results of the sensitivity analysis emphasize that the concentration of pollutants is the most crucial factor, playing a key role in shaping the overall carcinogenic risk.

## 4. Conclusions

This study aimed to investigate the levels, sources, and health risks of OPEs and PBDEs present in vehicle dust. TDCiPP was the primary OPE detected, while BDE-209 was the major PBDE identified in the dust samples. This study used a combination of PMF modeling and correlation analyses to determine and quantify the sources of OPEs and PBDEs. Probabilistic risk estimation indicated that the noncarcinogenic and carcinogenic risks of exposure to OPEs and PBDEs through ingestion, dermal contact, and inhalation were relatively low for both occupational and nonoccupational populations. The findings of this study provide valuable information on the concentrations of OPEs and PBDEs in vehicle dust and their daily intake via different routes. This information can help support the prevention and risk control of OPE and PBDE pollution from vehicle dust.

## Figures and Tables

**Table 1:** Mean and range concentrations (ng g^−1^) of OPEs and PBDEs in vehicle dust samples.

Compound	Mean	Range	SD	MDL ^1^
TnBP	1.91 × 10^2^	2.00 × 10^−1^–1.40 × 10^4^	3.89 × 10^2^	5.00 × 10^−2^
TBOEP	9.81 × 10^3^	7.40 × 10^−2^–3.60 × 10^5^	1.74 × 10^4^	7.40 × 10^−2^
TEHP	5.88 × 10^2^	1.20 × 10^−1^–6.40 × 10^4^	8.05 × 10^2^	1.20 × 10^−1^
TCEP	5.38 × 10^3^	7.20 × 10^−2^–2.45 × 10^5^	9.87 × 10^3^	7.20 × 10^−2^
TCiPP	9.47 × 10^3^	2.00 × 10–3.70 × 10^5^	1.76 × 10^4^	1.00 × 10^−1^
TDCiPP	4.34 × 10^4^	5.00–7.40 × 10^5^	1.04 × 10^5^	6.70 × 10^−2^
TPhP	6.06 × 10^3^	2.30 × 10^−1^–1.70 × 10^5^	1.79 × 10^4^	2.30 × 10^−1^
EHDPP	3.30 × 10^3^	7.80 × 10^−2^–2.40 × 10^5^	8.85 × 10^3^	7.80 × 10^−2^
∑_8_OPEs	9.78 × 10^3^	2.58 × 10–2.20 × 10^6^	4.42 × 10^4^	
BDE-28	1.14 × 10	1.00 × 10^−2^–1.49 × 10^3^	1.28 × 10	3.00 × 10^−2^
BDE-47	4.41 × 10^2^	1.00 × 10^−2^–1.02 × 10^5^	1.24 × 10^3^	3.00 × 10^−3^
BDE-99	3.48 × 10^2^	1.00 × 10^−2^–2.25 × 10^5^	5.45 × 10^2^	3.00 × 10^−4^
BDE-100	1.11 × 10^2^	2.00 × 10^−2^–9.82 × 10^4^	2.72 × 10^2^	3.00 × 10^−3^
BDE-153	1.01 × 10^2^	1.00 × 10^−2^–1.79 × 10^4^	2.15 × 10^2^	1.00 × 10^−3^
BDE-154	4.07 × 10	1.00 × 10^−2^–6.67 × 10^4^	9.42 × 10	3.00 × 10^−3^
BDE-183	9.27 × 10	1.00 × 10^−2^–1.28 × 10^3^	2.33 × 10^2^	1.00 × 10^−2^
BDE-209	1.52 × 10^4^	1.00 × 10^−2^–2.61 × 10^5^	2.28 × 10^4^	9.39 × 10^−2^
∑_8_PBDEs	2.04 × 10^3^	9.00 × 10^−2^–7.73 × 10^5^	9.18 × 10^3^	

1: These MDLs are the minimum values from the literature we selected.

**Table 2:** ADDs (ng kg^−1^ day^−1^) for targeted OPEs and PBDEs via ingestion, dermal absorption, and inhalation.

	Occupational Populations			Nonoccupational Populations		
	ADD_ing_	ADD_inh_	ADD_der_	ADD_ing_	ADD_inh_	ADD_der_
TnBP	3.65 × 10^−8^	1.07 × 10^−14^	6.75 × 10^−9^	5.50 × 10^−9^	1.62 × 10^−15^	1.02 × 10^−9^
TBOEP	1.87 × 10^−6^	5.51 × 10^−13^	3.79 × 10^−7^	2.82 × 10^−7^	8.30 × 10^−14^	5.70 × 10^−8^
TEHP	1.12 × 10^−7^	3.30 × 10^−14^	2.27 × 10^−8^	1.69 × 10^−8^	4.98 × 10^−15^	3.42 × 10^−9^
TCEP	1.03 × 10^−6^	3.03 × 10^−13^	2.69 × 10^−7^	1.55 × 10^−7^	4.56 × 10^−14^	4.05 × 10^−8^
TCiPP	1.81 × 10^−6^	5.32 × 10^−13^	4.58 × 10^−7^	2.72 × 10^−7^	8.01 × 10^−14^	6.90 × 10^−8^
TDCiPP	8.29 × 10^−6^	2.44 × 10^−12^	9.72 × 10^−7^	1.25 × 10^−6^	3.67 × 10^−13^	1.46 × 10^−7^
TPhP	1.16 × 10^−6^	3.41 × 10^−13^	2.34 × 10^−7^	1.74 × 10^−7^	5.13 × 10^−14^	3.52 × 10^−8^
EHDPP	6.32 × 10^−7^	1.86 × 10^−13^	1.28 × 10^−7^	9.51 × 10^−8^	2.80 × 10^−14^	1.92 × 10^−8^
∑_8_OPEs	1.49 × 10^−5^	4.40 × 10^−12^	2.47 × 10^−6^	2.25 × 10^−6^	6.62 × 10^−13^	3.72 × 10^−7^
BDE-28	2.19 × 10^−9^	6.43 × 10^−16^	2.02 × 10^−10^	3.29 × 10^−10^	9.68 × 10^−17^	2.02 × 10^−10^
BDE-47	8.44 × 10^−8^	2.48 × 10^−14^	7.79 × 10^−9^	1.27 × 10^−8^	3.73 × 10^−15^	7.79 × 10^−9^
BDE-99	6.65 × 10^−8^	1.96 × 10^−14^	6.14 × 10^−9^	1.00 × 10^−8^	2.95 × 10^−15^	6.14 × 10^−9^
BDE-100	2.12 × 10^−8^	6.24 × 10^−15^	1.96 × 10^−9^	3.19 × 10^−9^	9.39 × 10^−16^	1.96 × 10^−9^
BDE-153	1.94 × 10^−8^	5.70 × 10^−15^	1.79 × 10^−9^	2.92 × 10^−9^	8.59 × 10^−16^	1.79 × 10^−9^
BDE-154	7.78 × 10^−9^	2.29 × 10^−15^	7.18 × 10^−10^	1.17 × 10^−9^	3.44 × 10^−16^	7.18 × 10^−10^
BDE-183	1.77 × 10^−8^	5.21 × 10^−15^	1.64 × 10^−9^	2.67 × 10^−9^	7.85 × 10^−16^	1.64 × 10^−9^
BDE-209	2.90 × 10^−6^	8.52 × 10^−13^	2.67 × 10^−7^	4.36 × 10^−7^	1.28 × 10^−13^	2.67 × 10^−7^
∑_8_PBDEs	3.12 × 10^−6^	9.17 × 10^−13^	2.88 × 10^−7^	4.69 × 10^−7^	1.38 × 10^−13^	4.33 × 10^−8^

## Data Availability

The datasets used and/or analyzed during the current study are available from the corresponding author on reasonable request.

## Supplementary Materials

The following supporting information can be downloaded at https://www.mdpi.com/article/10.3390/toxics12110806/s1. The Supplementary Materials include the following content: Table S1: Data sources and related information. Table S2: Exposure parameters for occupational and nonoccupational populations with references. Table S3: Rotated component matrix for OPEs. Table S4: Rotated component matrix for PBDEs. Table S5: HI and CR values for OPEs and PBDEs via ingestion, dermal absorption, and inhalation. Table S6: Statistics of probabilistic estimation of lifetime carcinogenic risk values. Figure S1: Scree plots for OPEs. Figure S2: Scree plots for PBDEs.

## Abbreviations

ABS	Acrylonitrile–butadiene–styrene
*ADD*	The average daily dose
*AF*	The dermal absorption factor
*AT*	Average exposure time
*BW*	Average body weight
*C*	The measured dust concentration
*CF*	Conversion factor
*CR*	Cancer risks
*DA*	The adherence to skin
*ED*	Exposure duration
*EF*	Exposure frequency
*ET*	Exposure time
HI	Hazard index
HIPS	High-impact polystyrene
HQ	Hazard quotient
OPEs	Organophosphates
PBDEs	Polybrominated diphenyl ethers
PCA	Principal component analysis
PE	Polyethylene
PEF	Particle emission factor
PMF	Positive matrix factorization
PP	Polypropylene
PUF	Polyurethane foam
PVC	Polyvinyl chloride
*R_ing_*	Ingestion rate
*R_inh_*	Inhalation rate
RfD	Reference concentration
*SA*	The exposure of skin
*SF*	Slope factor
