# Supplementary material for: Organophosphate Esters and Polybrominated Diphenyl Ethers in Vehicle Dust: Concentrations, Sources, and Health Risk Assessment
Source: Toxics. 2024 Nov 7;12(11):806. doi: 10.3390/toxics12110806 (PMC11598807; doi:10.3390/toxics12110806)
Supplement: Supplementary file 1 [file toxics-12-00806-s001.zip › toxics-3240080-supplementary.pdf]

Organophosphate esters and polybrominated diphenyl ethers in vehicle dust: Concentrations, sources and health risk assessment

Junji Wang [1,2], Jianzai Lin [1,2], Xi Zhang [3], Qinghong Zeng [1,2], Zhu Zhu [1,2], Siyuan Zhao [1,2], Deyan Cao [1,2] and Meilin Zhu [1,3] *

1 School of Public Health, Ningxia Medical University, Yinchuan 750004, China; wjjeee2022@163.com

2 Key Laboratory of Environmental Factors and Chronic Disease Control, Ningxia Medical University, Yinchuan 750004, China

3 School of Basic Medical Sciences, Ningxia Medical University, Yinchuan 750004, China

Supporting information

Table S1. Data sources and related information.

Type	Number of samples	Sampling Year	Country	References
OPEs	14	2017-2018	Greece	[1]
	30	-	Greece	[2]
	100	-	China	[3]
	15	-	Kingdom of Saudi Arabia	[4]
	25	-	Greece	[5]

	15	-	Kuwait	[6]
	15	-	Pakistan	[6]
	36	-	Egypt	[7]
	19	2013	South Africa	[8]
	15	2015	Germany	[9]
	20	2012-2013	Egypt	[10]
	21	2011-2012	England	[11]
	15	2015	Australia	[12]
	4	-	China	[13]
	12	2010-2011	Germany	[14]
	8	2012	Netherlands	[15]
	10	2021	Nigeria	[16]
PBDEs	8	-	Australia	[17]
	20	2019-2020	China	[18]
	12	2014	Egypt	[19]
	15	2019	China	[20]
	66	-	American	[21]
	20	-	Nigeria	[22]
	30	2007-2008	Thailand	[23]
	16	2014	Nigeria	[24]
	12	2014	Nigeria	[25]
	40	2018-2019	Kuwait	[26]

	15	2019	Nigeria	[27]
	30	-	Greece	[2]
	19	2011	Kuwait	[28]

Table S2. Exposure parameters for occupational and non-occupational populations with references.

Abbreviations	Exposure Factors	Exposure Values	References
C	The measured dust concentration (ng g^{-1})	Shown in Table 1	Shown in Table S1
R_{ing}	Ingestion rate (g day^{-1})	0.05	[29]
R_{inh}	Inhalation rate（m^3 day^{-1}）	20	[30]
ET	Exposure time（hours day^{-1}）	0.279 for occupational populations 0.042 for nonoccupational populations	[29]
EF	Exposure frequency（days $year^{-1}$）	350	[5]
ED	Exposure duration	30	[5]

	(years)		
PEF	Particle emission factor (m^3 kg^{-1})	1.36 × 10^9	[31]
SA	The exposure skin area (cm^2)	4615	[32]
DA	The adhered to skin (mg cm^{-2})	0.01	[31]
AF	The dermal absorption factor (unitless)	0.219 for TEHP, TBEP, TPHP, EHDPP<br>0.283 for TCEP<br>0.127for TDCIPP<br>0.274 for TCIPP<br>0.200 for TNBP<br>0. 100 for PBDEs	[31]
BW	Average body weight (kg)	70	[5]
AT	Average exposure time (days)	30 × 365<br>70 × 365	[31]
CF	Conversion factor	10^{-6}	-
RfD	Reference concentration (mg kg^{-1} day^{-1})	0.024 for TNBP<br>0.015 for TBEP and TDCIPP	[31]

		0.022 for TCEP<br>0.08 for TCIPP<br>0.07 for TPHP<br>0.0001 for BDE-47 and BDE-99<br>0.0002 for BDE-153<br>0.007 for BDE-209	
SF	Slope factor	0.009 for TNBP<br>0.0032 for TEHP<br>0.02 for TCEP<br>0.031 for TDCIPP<br>0.0007 for BDE-209	[31]

Table S3.Concentration (ng g^{-1}) of OPEs in vehicle dust in different countries

	China	Kuwait	Pakistan	Egypt	South Africa	Germany	England	Greece	Nigeria	Australia	Kingdom of Saudi Arabia	Netherlands
TnBP	1.00E+02	1.69E+03	5.00E+01	4.95E+01	-	2.60E+02	1.40E+02	4.36E+02	2.60E+01	1.70E+02	5.40E+02	4.35E+01
TBOEP	3.60E+03	5.27E+03	1.25E+02	2.16E+02	-	1.43E+04	-	3.70E+02	9.90E+01	4.60E+04	1.65E+03	2.65E+04
TEHP	2.90E+03	2.12E+02	4.70E+01	1.20E-01	-	2.00E+03	-	6.37E+03	-	4.50E+02	1.95E+02	-
TCEP	1.66E+04	3.48E+03	2.55E+02	1.15E+02	3.51E+04	1.18E+03	1.95E+03	3.39E+03	8.20E+00	1.20E+03	7.02E+03	1.70E+03
TCiPP	1.16E+04	3.60E+04	5.90E+02	3.56E+02	1.01E+04	5.45E+03	8.30E+04	2.50E+03	7.10E+01	2.60E+04	1.63E+04	5.00E+03
TDCiPP	9.00E+02	3.58E+04	1.30E+02	1.30E+02	8.92E+04	6.74E+04	1.10E+05	9.98E+02	1.80E+01	1.50E+03	8.85E+03	6.35E+04
TPhP	-	2.17E+03	6.65E+02	2.69E+02	9.33E+03	2.60E+03	1.50E+04	1.06E+03	1.90E+01	6.70E+03	7.86E+02	2.05E+03
EHDPP	2.20E+03	8.75E+02	7.30E+01	3.30E+01	-	-	2.90E+03	0.00E+00	3.70E+01	3.90E+04	1.05E+03	1.13E+03
$\sum_8$OPEs	3.79E+04	8.54E+04	1.94E+03	1.17E+03	1.44E+05	9.31E+04	2.13E+05	1.51E+04	2.78E+02	1.21E+05	3.63E+04	9.99E+04

Note: "-" indicates that the substance was not detected.

Table S4.Concentration (ng g^{-1}) of PBDEs in vehicle dust in different countries

	China	Germany	Kuwait	Nigeria	Thailand	Pakistan	American	Egypt	Greece	Australia
BDE-28	2.69E+01	-	3.90E+00	1.17E+01	3.20E-01	2.00E-01	1.32E+01	3.75E+00	1.22E+01	-
BDE-47	6.28E+01	1.70E+01	1.64E+01	2.53E+02	1.80E+00	1.80E+00	1.87E+03	4.65E+01	1.81E+02	1.40E+02
BDE-99	1.36E+02	3.20E+01	2.00E+01	4.82E+02	3.00E+00	2.50E+00	9.22E+02	5.80E+01	3.43E+02	1.80E+02
BDE-100	9.58E+01	-	2.11E+00	1.08E+02	4.50E+01	4.00E-01	3.44E+02	1.38E+01	4.31E+01	4.00E+01
BDE-153	8.58E+01	-	3.39E+00	2.06E+02	4.50E-01	1.00E+00	2.22E+02	3.45E+01	5.86E+01	1.70E+01
BDE-154	6.67E+01	-	1.52E+00	1.34E+02	2.30E-01	4.00E-01	2.31E+01	8.97E+00	3.67E+01	1.20E+01
BDE-183	5.26E+01	3.70E+00	2.43E+00	4.88E+01	-	3.00E+00	4.46E+00	1.09E+01	3.04E+02	-
BDE-209	1.95E+03	9.40E+02	5.43E+03	5.07E+03	-	3.01E+04	3.72E+04	6.81E+03	1.31E+04	1.40E+04
$\sum_8$PBDEs	2.48E+03	9.93E+02	5.48E+03	6.31E+03	5.08E+01	3.01E+04	4.06E+04	6.99E+03	1.41E+04	1.44E+04

Note: "-" indicates that the substance was not detected.

Table S5. Rotated component matrix for OPEs.

OPEs	F1	F2	F3	F4
TnBP	-0.068	-0.020	**0.997**	-0.004
TBOEP	**0.793**	0.529	-0.073	0.015
TEHP	0.003	-0.004	-0.004	**1.000**

TDCiPP	**0.941**	-0.127	-0.030	-0.012
TPhP	**0.954**	0.073	-0.052	0.009
EHDPP	0.027	**0.983**	-0.011	-0.007
Variance (%)	40.510	21.145	16.732	16.673

Table S6. Rotated component matrix for PBDEs.

PBDEs	F1	F2	F3
BDE-28	0.593	-0.148	-0.295
BDE-47	**0.933**	-0.037	0.090
BDE-99	**0.869**	0.329	0.277
BDE-100	**0.964**	-0.013	0.019
BDE-153	**0.909**	0.356	0.027
BDE-154	0.459	**0.710**	-0.011
BDE-183	-0.130	**0.760**	-0.060
BDE-209	0.061	-0.090	**0.956**
Variance (%)	49.577	16.854	13.636

Note: extraction method based on principal component analysis; a: bold is the main load.

Table S7. HI and CR values for OPEs and PBDEs via ingestion, dermal absorption, and inhalation.

	Occupational populations			Nonoccupational populations		
	HQ_{ing}	HQ_{inh}	HQ_{der}	HQ_{ing}	HQ_{inh}	HQ_{der}
TnBP	1.52E-06	4.48E-13	2.81E-07	2.29E-07	6.74E-14	4.23E-08

TBOEP	1.25E-04	3.68E-11	2.53E-05	1.88E-05	5.53E-12	3.80E-06
TCEP	4.68E-05	1.38E-11	1.22E-05	7.04E-06	2.07E-12	1.84E-06
TCiPP	2.26E-05	6.65E-12	5.72E-06	3.41E-06	1.00E-12	8.61E-07
TDCiPP	5.53E-04	1.63E-10	6.48E-05	8.32E-05	2.45E-11	9.75E-06
TPhP	1.65E-05	4.87E-12	3.34E-06	2.49E-06	7.33E-13	5.03E-07
BDE-47	8.44E-04	2.48E-10	7.79E-05	1.27E-04	3.73E-11	1.17E-05
BDE-99	6.65E-04	1.96E-10	6.14E-05	1.00E-04	2.95E-11	9.25E-06
BDE-153	9.70E-05	2.85E-12	8.95E-06	1.46E-05	4.29E-12	1.35E-06
BDE-209	4.14E-04	1.22E-10	3.82E-05	6.23E-05	1.83E-11	5.75E-06
	HI_{ing}	HI_{inh}	HI_{der}	HI_{ing}	HI_{inh}	HI_{der}
	2.79E-03	8.20E-10	2.98E-04	4.19E-04	1.23E-10	4.48E-05
	CR_{ing}	CR_{inh}	CR_{der}	CR_{ing}	CR_{inh}	CR_{der}
TnBP	1.41E-10	4.15E-17	2.60E-11	2.12E-11	6.24E-18	3.92E-12
TEHP	1.54E-10	4.53E-17	2.84E-11	2.32E-11	6.82E-18	4.69E-12
TCEP	8.82E-09	2.59E-15	1.63E-09	1.33E-09	3.91E-16	3.47E-10
TDCiPP	1.10E-07	3.24E-14	2.03E-08	1.66E-08	4.88E-15	1.94E-09
BDE-209	8.69E-10	2.56E-16	8.02E-11	1.31E-10	3.85E-17	1.21E-11
	TCR_{ing}	TCR_{inh}	TCR_{der}	TCR_{ing}	TCR_{inh}	TCR_{der}
	1.20E-07	3.53E-14	2.21E-08	1.81E-08	5.32E-15	2.31E-09

Table S8. Statistics of probabilistic estimation of lifetime carcinogenic risk values.

	Group	Distribution	Parameters		10%	50%	90%
			Mean	SD			
HI	Occupation	Lognormal	3.14×10^{-3}	3.60×10^{-3}	1.00×10^{-3}	2.28×10^{-3}	5.85×10^{-3}
	Nonoccupation	Lognormal	3.34×10^{-4}	5.25×10^{-4}	1.52×10^{-4}	3.43×10^{-4}	8.80×10^{-4}
CR	Occupation	Lognormal	1.39×10^{-7}	2.80×10^{-7}	1.52×10^{-8}	5.98×10^{-8}	3.00×10^{-7}
	Nonoccupation	Lognormal	2.08×10^{-8}	4.23×10^{-8}	2.33×10^{-9}	9.11×10^{-9}	4.63×10^{-8}

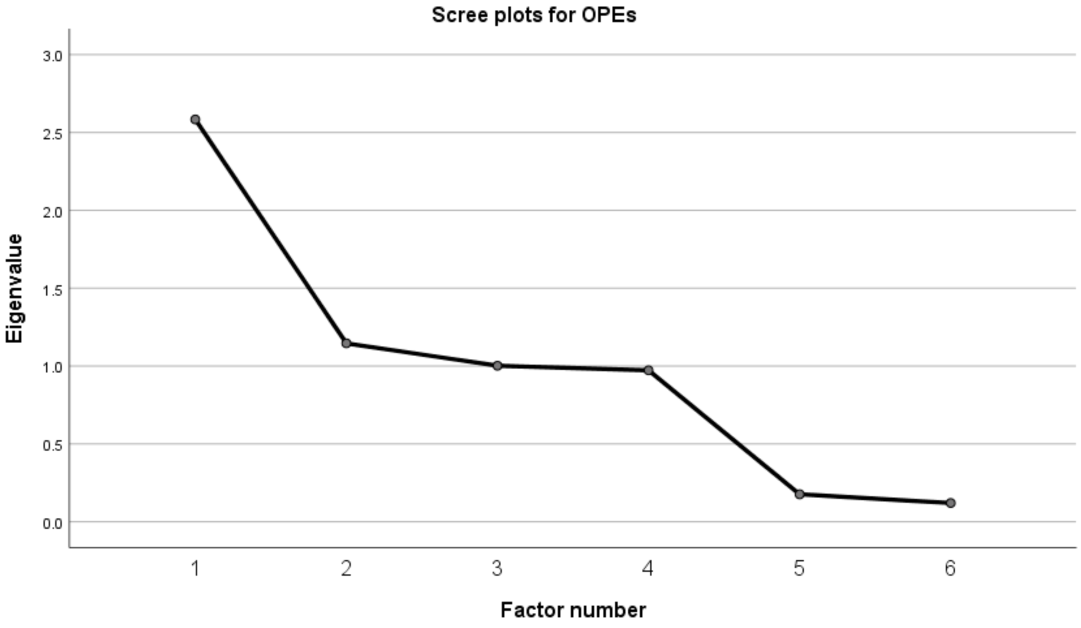

Figure S1. Scree plots for OPEs.

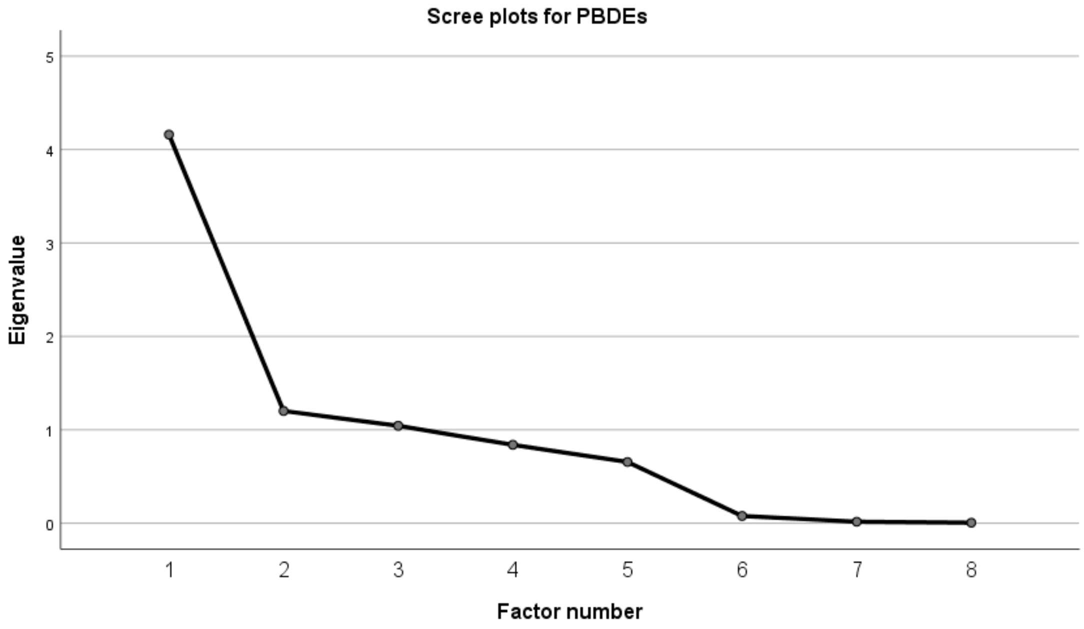

Figure S2. Scree plots for PBDEs.

References

[1] Petromelidou S, Margaritis D, Nannou C, et al. HRMS screening of organophosphate flame retardants and poly-/perfluorinated substances in dust from cars and trucks: occurrence and human exposure implications[J]. Science of the Total Environment, 2022, 848: 157696.

[2] Besis A, Christia C, Poma G, et al. Legacy and novel brominated flame retardants in interior car dust - implications for human exposure[J]. Environmental Pollution (Barking, Essex: 1987), 2017, 230: 871-881.

[3] He R W, Li Y Z, Xiang P, et al. Impact of particle size on distribution and human exposure of flame retardants in indoor dust[J]. Environmental Research, 2018, 162: 166-172.

[4] Ali N, Eqani S A M A S, Ismail I M I, et al. Brominated and organophosphate flame retardants in indoor dust of jeddah, kingdom of saudi arabia: implications for human exposure[J]. Science of the Total Environment, 2016, 569-570: 269-277.

[5] Christia C, Poma G, Besis A, et al. Legacy and emerging organophosphorus flame retardants in car dust from greece: implications for human exposure[J]. Chemosphere, 2018, 196: 231-239.

[6] Ali N, Ali L, Mehdi T, et al. Levels and profiles of organochlorines and flame retardants in car and house dust from kuwait and pakistan: implication for human exposure via dust ingestion[J]. Environment International, 2013, 55: 62-70.

[7] Khairy M A, Lohmann R. Organophosphate flame retardants in the indoor and outdoor dust and gas-phase of alexandria, egypt[J]. Chemosphere, 2019, 220: 275-285.

[8] Abafe O A, Martincigh B S. Concentrations, sources and human exposure implications of organophosphate esters in indoor dust from south africa[J]. Chemosphere, 2019, 230: 239-247.

[9] Occurrence and human exposure assessment of organophosphate flame retardants in indoor dust from various microenvironments of the rhine/main region, germany - PubMed[EB/OL]. [2024-06-11]. https://pubmed.ncbi.nlm.nih.gov/28556503/.

[10] Abdallah M A E, Covaci A. Organophosphate flame retardants in indoor dust from Egypt: implications for human exposure[J]. Environmental Science & Technology, 2014, 48(9): 4782-4789.

[11] Brommer S, Harrad S. Sources and human exposure implications of concentrations of organophosphate flame retardants in dust from UK cars, classrooms, living rooms, and offices[J]. Environment International, 2015, 83: 202-207.

[12] He C, Wang X, Thai P, et al. Organophosphate and brominated flame retardants in australian indoor environments: levels, sources, and preliminary assessment of human exposure[J]. Environmental Pollution (Barking, Essex: 1987), 2018, 235: 670-679.

[13] Peng C, Tan H, Guo Y, et al. Emerging and legacy flame retardants in indoor dust from east China[J]. Chemosphere, 2017, 186: 635-643.

[14] Brommer S, Harrad S, Van den Eede N, et al. Concentrations of organophosphate esters and brominated flame retardants in german indoor dust samples[J]. Journal of

environmental monitoring: JEM, 2012, 14(9): 2482-2487.
[15] Brandsma S H, de Boer J, van Velzen M J M, 等. Organophosphorus flame retardants (PFRs) and plasticizers in house and car dust and the influence of electronic equipment[J]. Chemosphere, 2014, 116: 3-9.
[16] Gbadamosi M R, Ogunneye A L, Al-Omran L S, et al. Presence, source attribution, and human exposure to organophosphate esters in indoor dust from various microenvironments in Nigeria[J]. Emerging Contaminants, 2023, 9(2): 100208.
[17] McGrath T J, Morrison P D, Ball A S, et al. Concentrations of legacy and novel brominated flame retardants in indoor dust in melbourne, australia: an assessment of human exposure[J]. Environment International, 2018, 113: 191-201.
[18] Jin M, Ye N, Lu Z, et al. Pollution characteristics and source identification of PBDEs in public transport microenvironments[J]. Science of the Total Environment, 2022, 820: 153159.
[19] Khairy M A, Lohmann R. Selected organohalogenated flame retardants in Egyptian indoor and outdoor environments: Levels, sources and implications for human exposure[J]. Science of The Total Environment, 2018, 633: 1536-1548.
[20] Polybrominated diphenyl ethers from automobile microenvironment: occurrence, sources, and exposure assessment - ScienceDirect[EB/OL]. [2024-06-11]. https://www.sciencedirect.com/science/article/abs/pii/S0048969721017265.
[21] Lagalante A F, Shedden C S, Greenbacker P W. Levels of polybrominated diphenyl ethers (PBDEs) in dust from personal automobiles in conjunction with studies on the photochemical degradation of decabromodiphenyl ether (BDE-209)[J]. Environment International, 2011, 37(5): 899-906.
[22] Adeyi A A, Akanmu F R, Babalola B A, et al. Levels of polybrominated diphenyl ethers (PBDEs) in indoor dusts in lagos and ibadan, nigeria[J]. Microchemical Journal, 2020, 158: 105132.
[23] Muenhor D, Harrad S. Polybrominated diphenyl ethers (PBDEs) in car and house dust from Thailand: implication for human exposure[J]. Journal of Environmental Science and Health. Part A, Toxic/Hazardous Substances and Environmental Engineering, 2018, 53(7): 629-642.
[24] Harrad S, Abdallah M A E, Oluseyi T. Polybrominated diphenyl ethers and polychlorinated biphenyls in dust from cars, homes, and offices in lagos, nigeria[J]. Chemosphere, 2016, 146: 346-353.
[25] Olukunle O I, Okonkwo O J, Wase A G, et al. Polybrominated diphenyl ethers in car dust in Nigeria: concentrations and implications for non-dietary human exposure[J]. Microchemical Journal, 2015, 123: 99-104.
[26] Human health risks from brominated flame retardants and polycyclic aromatic hydrocarbons in indoor dust - ScienceDirect[EB/OL]. [2024-06-11]. https://www.sciencedirect.com/science/article/abs/pii/S0045653521014776.
[27] Ibeto C, Aju E, Imafidon B, et al. Exposure evaluation and risk assessment of polybrominated diphenyl ethers in dust from microenvironments in nsukka, nigeria[J]. Environmental Science and Pollution Research International, 2021.
[28] Gevao B, Shammari F, Ali L N. Polybrominated diphenyl ether levels in dust collected from cars in Kuwait: Implications for human exposure[J]. Indoor and Built

Environment, 2014.
[29] Thuresson K, Björklund J A, de Wit C A. Tri-decabrominated diphenyl ethers and hexabromocyclododecane in indoor air and dust from Stockholm microenvironments 1: Levels and profiles[J]. Science of The Total Environment, 2012, 414: 713-721.
[30] Ali N, Dirtu A C, Van den Eede N, et al. Occurrence of alternative flame retardants in indoor dust from new zealand: indoor sources and human exposure assessment[J]. Chemosphere, 2012, 88(11): 1276-1282.
[31] Us Epa O. Regional screening levels (RSLs) - generic tables[EB/OL]. (2015-09-03)[2024-04-05]. https://www.epa.gov/risk/regional-screening-levels-rsls-generic-tables.
[32] Abdallah M A E, Harrad S. Personal exposure to HBCDs and its degradation products via ingestion of indoor dust[J]. Environment International, 2009, 35(6): 870-876.
